# Cancer Mortality by Country of Birth, Sex, and Socioeconomic Position in Sweden, 1961–2009

**DOI:** 10.1371/journal.pone.0093174

**Published:** 2014-03-28

**Authors:** Gholamreza Abdoli, Matteo Bottai, Tahereh Moradi

**Affiliations:** 1 Division of Epidemiology, Institute of Environmental Medicine, Karolinska Institutet, Stockholm, Sweden; 2 Department of Biostatistics and Epidemiology, Kermanshah University of Medical Sciences, Kermanshah, Iran; 3 Unit of Biostatistics, Institute of Environmental Medicine, Karolinska Institutet, Stockholm, Sweden; 4 Centrum for Epidemiology and Social Medicine, Healthcare Provision, Stockholm, Sweden; Universität Bochum, Germany

## Abstract

In 2010, cancer deaths accounted for more than 15% of all deaths worldwide, and this fraction is estimated to rise in the coming years. Increased cancer mortality has been observed in immigrant populations, but a comprehensive analysis by country of birth has not been conducted. We followed all individuals living in Sweden between 1961 and 2009 (7,109,327 men and 6,958,714 women), and calculated crude cancer mortality rates and age-standardized rates (ASRs) using the world population for standardization. We observed a downward trend in all-site ASRs over the past two decades in men regardless of country of birth but no such trend was found in women. All-site cancer mortality increased with decreasing levels of education regardless of sex and country of birth (p for trend <0.001). We also compared cancer mortality rates among foreign-born (13.9%) and Sweden-born (86.1%) individuals and determined the effect of education level and sex estimated by mortality rate ratios (MRRs) using multivariable Poisson regression. All-site cancer mortality was slightly higher among foreign-born than Sweden-born men (MRR = 1.05, 95% confidence interval 1.04–1.07), but similar mortality risks was found among foreign-born and Sweden-born women. Men born in Angola, Laos, and Cambodia had the highest cancer mortality risk. Women born in all countries except Iceland, Denmark, and Mexico had a similar or smaller risk than women born in Sweden. Cancer-specific mortality analysis showed an increased risk for cervical and lung cancer in both sexes but a decreased risk for colon, breast, and prostate cancer mortality among foreign-born compared with Sweden-born individuals. Further studies are required to fully understand the causes of the observed inequalities in mortality across levels of education and countries of birth.

## Introduction

Worldwide, in 2010 there were 8 million deaths due to cancer, accounting for 15.1% of all deaths [1]. It has been estimated that this figure may rise to 13 million deaths by 2030 [2]. According to a report published by the World Health Organization, cancers of the lung, stomach, liver, colon and breast are the leading causes of cancer deaths [2]. Furthermore, 20% of all deaths in Europe are due to cancer, with more than 1.7 million annual deaths [3]. Cancer is the second most common cause of mortality in Sweden (26% of deaths among men and 22% among women) [4] and contributes to 60% of the total burden of disease [5]. Among the different cancer types, lung cancer is the most common cause of cancer death in women, whereas prostate cancer is the most common cause of cancer death in men [4]. Mortality due to cancer has been shown to be markedly higher among immigrants than non-immigrants in a number of countries [6], [7], [8]. Because of the increasing number of transnational migrants, estimated at 3.1% of the global population in 2010 [9], focused research is needed among immigrant populations worldwide to investigate, for example, the etiology of diseases, gene–environment interactions, potential inequalities with respect to access to healthcare services, and treatments of interest from a healthcare perspective to better optimize the use of resources. In addition, useful information regarding the impact of environmental factors on mortality could be gained through investigation of cancer mortality among foreign-born individuals [10].

We evaluated the risks of mortality due to all-site cancer and to some common specific (colon, lung, stomach, prostate, breast and cervical) cancers in the total Swedish population and in subgroups of the large and currently increasing foreign-born population by individual birth country, sex, and socioeconomic position (SEP).

## Materials and Methods

### Ethics statement

According Swedish law, data recording in national health registers, such as the Patient Register and the Cancer Register, does not require consent from either patients or healthcare providers. It is mandatory to report to these registers, and patients cannot refuse to allow registration of their data. Ethical vetting is always required when using register data for research in Sweden. This is performed by regional ethical review boards and the risk appraisal associated with the Law on Public Disclosure and Secrecy is carried out by the data holders. The ethical review boards can however waive the requirement to consult the data subjects directly to obtain their informed consent. According to these standards, the Regional Ethical Review Board in Stockholm, Sweden has waived the requirement to consult the data subjects directly to obtain their informed consent for this project and has evaluated and approved the study.

### Data

We conducted a nationwide cohort study between 1961 and 2009 using the Migration and Health Cohort that was built by linkage of several Swedish national demographic and health registers through the 10-digit unique Swedish personal identification number. The Migration and Health Cohort was created to specifically address health status among immigrants and their offspring in Sweden. The data used in this study are from this cohort and include the following five registers. 1) The Immigration Register contains data on immigration and country of birth. 2) The Register of the Total Population at Statistics Sweden contains demographic information, country of birth and data on emigration and immigration. This register was officially initiated in 1968; between 1961 and 1967 population registration was based on printed cards sent from the county administrative boards to Statistics Sweden [11]. 3) The Cause of Death Register contains data on the date and the main cause of death from 1961, and is updated yearly [12]. This register is based on the International Classification of Diseases (ICD), 7^th^, 8^th^, 9^th^, and 10^th^ revisions [13]. The non-reporting rate has been estimated at less than 2% [4], [14]. In our cohort the proportion of missing data on cause of death among individuals who had died was 0.04% overall for the entire study period (1961–2009), 0% during the period 1961–1990, 0.19% during 1991–2000, and 0% for 2001–2009. 4) The Swedish Population and Housing Census contain sociodemographic data including country of birth from 1960 to 1990. 5) Finally, the longitudinal integration database for health insurance and labor market studies contains information on the highest attained level of education since 1990 [13], [15]. The level of education is updated annually from many different sources, most of which are other registers at Statistics Sweden or other authorities. Since 2000, a questionnaire has been sent to foreign-born residents with an unknown level of education to request this information [16].

This study was approved by the Regional Board of The Ethics Committee of Stockholm (Dnr. 2009/2033-32).

### Study cohort

The study cohort consisted of all individuals (7,109,327 men and 6,958,714 women) living in Sweden at any time between January 1, 1961 and December 31, 2009. The study cohort included two groups: those born outside Sweden, referred to as “foreign-born” individuals, and those born within Sweden, referred to as “Sweden-born” individuals. The foreign-born group consisted of 1,950,551 individuals (13.9%) and the Sweden-born group of 12,117,490 individuals (86.1%).

### Follow-up

The cohort was followed from the beginning of 1961, the date of birth for Sweden-born individuals, or the date of immigration for foreign-born individuals, whichever occurred last, until the date of death due to cancer (see Table 1 for ICD codes), the date of first emigration, or the end of follow-up (December 31, 2009), whichever occurred first.

**Table 1:** International Classification of Diseases (ICD) codes for cancer death according to calendar period of study.

	ICD-7 (1961–1968)	ICD-8 (1969–1986)	ICD-9 (1987–1996)	ICD-10 (1997–2009)
All-site cancers	140–209	140–209	140–208	C00–C97
Lung cancer	162–163	162–163	162–163	C33–C34
Colon cancer	153	153	153	C18
Stomach cancer	151	151	151	C16
Breast cancer	170	174	174	C50
Cervical cancer	171	180	180	C53
Prostate cancer	177	185	185	C61

ICD-7, -8, -9, and -10, International Classification of Diseases, 7^th^, 8^th^, 9^th^, and 10^th^ revisions.

### Classification of place of birth

We classified place of birth for foreign-born individuals into six continents, which were further subdivided into 19 world regions, as defined by the United Nations Population Division as follows: Africa (East, Central, North, Southern, and West Africa), Asia (East, South-Central, South-East, and Western Asia), Europe (Eastern, Northern, Southern, and Western Europe), Latin America (Caribbean, Central America, and South America), North America, and Oceania (Australia/New Zealand, Melanesia, and Micronesia/Polynesia). We further reported data for individual countries of birth in which five or more deaths occurred due to cancer.

### Classification of SEP

We used the highest attained education level as an indicator of SEP using census data before 1990 and the longitudinal integration database for health insurance and labor market studies from 1990 onwards. The level of education was divided into four categories: 0–9 years, 10–12 years, more than 12 years, and unknown.

### Statistical methods

We calculated all-site and site-specific cancer mortality rate ratio (MRR) with the 95% confidence interval (CI) as a measure of relative risk using Poisson regression models. In the multivariable models we adjusted for age at follow-up in 19 groups of 5-year intervals (0–4, 5–9, …, 85–89, 90+ years) and calendar period at baseline in 10 groups of 5-year intervals (1961–1965, …, 2006–2009). We also considered marital status as a potential confounding factor in the models; however, following adjustment estimates of the other regression coefficients did not change by more than 5%. Therefore marital status was not included in the final model.

In assessing the association between level of education and cancer mortality in the MRR analysis, we restricted our cohort to individuals aged 30 years or above. Because composition of the education groups might have changed over time, we further evaluated the effect of education on all-site cancer mortality separately by decades of calendar time (1961–1970, …, 2001–2009).

In addition, we calculated crude cancer mortality rates (MRs) by dividing the number of cases by the observed person-years. Age-standardized rates (ASRs) were further calculated using the world population as standard [17]. Here we report crude MRs and ASRs per 100,000 person-years.

In a sensitivity analysis, we calculated separately the proportion of individuals who were 100 years and older among Sweden-born and foreign-born individuals, to gain some understanding of the proportion of individuals who may have died without a registered date of death and to determine whether this proportion differs between the Sweden-born and foreign-born groups. (i.e. assuming that these very elderly persons will have died).

All analyses were performed for men and women separately. P-values of <0.05 were considered statistically significant. Analyses were conducted using SAS, version 9.2.

## Results

During 412,386 million person-years of follow-up between 1961 and 2009, there were 464,690 all-site cancer deaths among men (29,142 foreign-born men) and 417,258 among women (29,537 foreign-born women). Foreign-born men and women were on average approximately 5 years younger at the time of all-site cancer death compared with Sweden-born men and women, respectively (mean age±SD: foreign-born men, 66.7±12.7 years; Sweden-born men, 71.6±12.4 years; foreign-born women, 67.2±13.8 years, Sweden-born women, 71.0±13.4 years). The mean (±SD) number of years of residence in Sweden was 16.2±15.2 for foreign-born men and 18.4±16.3 for foreign-born women (data not shown in Tables). The proportions of individuals of 100 years of age and older were extremely low and almost identical among Sweden-born (0.17%) and foreign-born (0.15%) individuals.

Table 2 shows crude cancer MRs per 100,000 person-years among foreign-born and Sweden-born individuals by cancer type and sex. All-site cancer MRs were lower in women than in men in both Sweden-born and foreign-born groups. Compared with Sweden-born men and women, all-site cancer MRs were lower in their foreign-born counterparts.

**Table 2:** Crude cancer mortality rate (MR) per 100,000 person-years and 95% confidence interval (CI) in foreign-born and Sweden-born men and women by cancer types in Sweden, 1961–2009.

	Foreign-born individuals			Sweden-born individuals		
	Non-cases*	Cases	MR (95% CI)	Non-cases*	Cases	MR (95% CI)
Men						
All-site cancers	950 113	29 142	173.64 (171.65–175.64)	5 694 524	435 548	232.02 (231.33–232.71)
Colon cancer	977 370	1 885	11.23 (10.72–11.74)	6 096 549	33 523	17.85 (17.66–18.05)
Lung cancer	971 339	7 916	47.16 (46.13–48.20)	6 061 613	68 459	36.47 (36.19–36.74)
Stomach cancer	976 984	2 271	13.53 (12.97–14.08)	6 091 985	38 087	20.29 (20.08–20.49)
Prostate cancer	976 000	3 255	19.39 (18.73–20.06)	6 044 354	85 718	45.66 (45.35–45.96)
Women						
All-site cancers	941 759	29 537	162.58 (160.73–164.44)	5 599 697	387 721	204.36 (203.72–205.00)
Colon cancer	968 956	2 340	12.88 (12.35–13.40)	5 951 702	35 716	18.82 (18.63–19.02)
Lung cancer	967 455	3 841	21.14 (20.47–21.81)	5 953 473	33 945	17.89 (17.70–18.08)
Stomach cancer	969 457	1 839	10.12 (9.66–10.58)	5 962 539	24 879	13.11 (12.95–13.27)
Breast cancer	966 584	4 712	25.93 (25.19–26.67)	5 925 606	61 812	32.58 (32.32–32.83)
Cervical cancer	970 381	915	5.03 (4.71–5.36)	5 978 106	9 312	4.90 (4.80–5.00)

*Total number of studied individuals excluding those who died due to each type of cancer.

**Figure 1:**
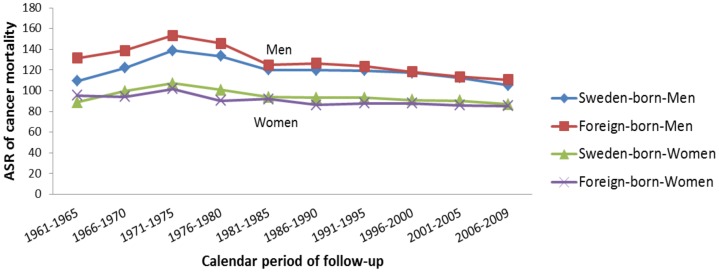
Age-standardized rate (ASR) of all-site cancer mortality by sex, calendar period of follow-up, and country of birth in Sweden, 1961–2009.

We observed an overall increasing trend in all-site cancer mortality over the period 1971–1975, regardless of sex and country of birth (Figure 1), and a decreasing trend during the remaining study period for men regardless of country of birth; no changes were observed for women in the past two decades (Figure 1).

**Figure 2:**
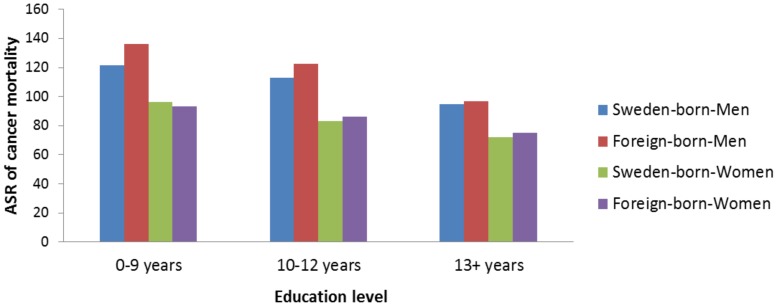
Age-standardized rate (ASR)* of all-site cancer mortality by sex, education level, and country of birth in Sweden, 1961–2009. *Only individuals who were ≥30 years old are included.

All-site cancer mortality decreased with increasing level of education irrespective of sex and country of birth (p for trend <0.0001) (Table 3). The same pattern was observed in the ASR analysis (Figure 2). Overall, the risk of all-site cancer mortality was higher among the least well educated compared with those with a higher level of education over the past few decades (data not shown). The risk was more prominent among foreign-born than Sweden-born men (foreign-born: MRR = 1.42, 95% CI 1.36–1.48; Sweden-born: MRR = 1.28, 95% CI 1.27–1.30) but in women, it was more noticeable among Sweden-born (Sweden-born: MRR = 1.39, 95% CI 1.37–1.41; foreign-born: MRR = 1.28, 95% CI 1.22–1.33) low versus high education level (Table 3). In the stratified analysis by calendar period, we found the same pattern of increased risk of all-site cancer mortality among individuals with a low level of education for all strata of calendar years, except for the years 1961–1970 when the association was reversed. Compared with highly educated individuals, mortality was decreased in those with a lower level of education, regardless of sex and country of birth (p for trend <0.0001) (data not shown).

**Table 3:** All-site cancer mortality rate ratio (MRR) and 95% confidence interval (CI) by country of birth, sex, and years of education in Sweden, 1961–2009.

	Sweden-born individuals			Foreign-born individuals		
	Cases	Person-years	MRR* (95% CI)	Cases	Person-years	MRR*(95% CI)
**Men**						
**Highest level of education (years)** **						
0–9	144 253	38 933 778	**1.28 (1.27–1.30)**	9 845	3 548 210	**1.42 (1.36–1.48)**
10–12	75 086	34 402 933	**1.19 (1.17–1.21)**	8 500	4 330 772	**1.26 (1.21–1.32)**
13+	30 898	18 624 416	1	3 426	2 491 613	1
P for trend			**<0.0001**			**<0.0001**
Unknown	181 026	17 331 776	**1.34 (1.32–1.36)**	7 100	1 205 782	**1.41 (1.35–1.47)**
**Women**						
**Highest level of education (years)** **						
0–9	138 252	40 500 445	**1.39 (1.37–1.41)**	11 068	4 530 404	**1.28 (1.22–1.33)**
10–12	56 846	33 111 786	**1.19 (1.17–1.21)**	6 931	4 238 821	**1.16 (1.11–1.22)**
13+	21 869	19 403 289	1	2 617	2 539 887	1
P for trend			**<0.0001**			**<0.0001**
Unknown	167 605	22 349 548	**1.48 (1.46–1.50)**	8 665	1 591 307	**1.33 (1.27–1.40)**

*MRR values significantly different from 1.0 are highlighted in bold.

** Only individuals ≥30 years old are included and MRRs are adjusted for age at follow-up and calendar period at baseline.

**Figure 3:**
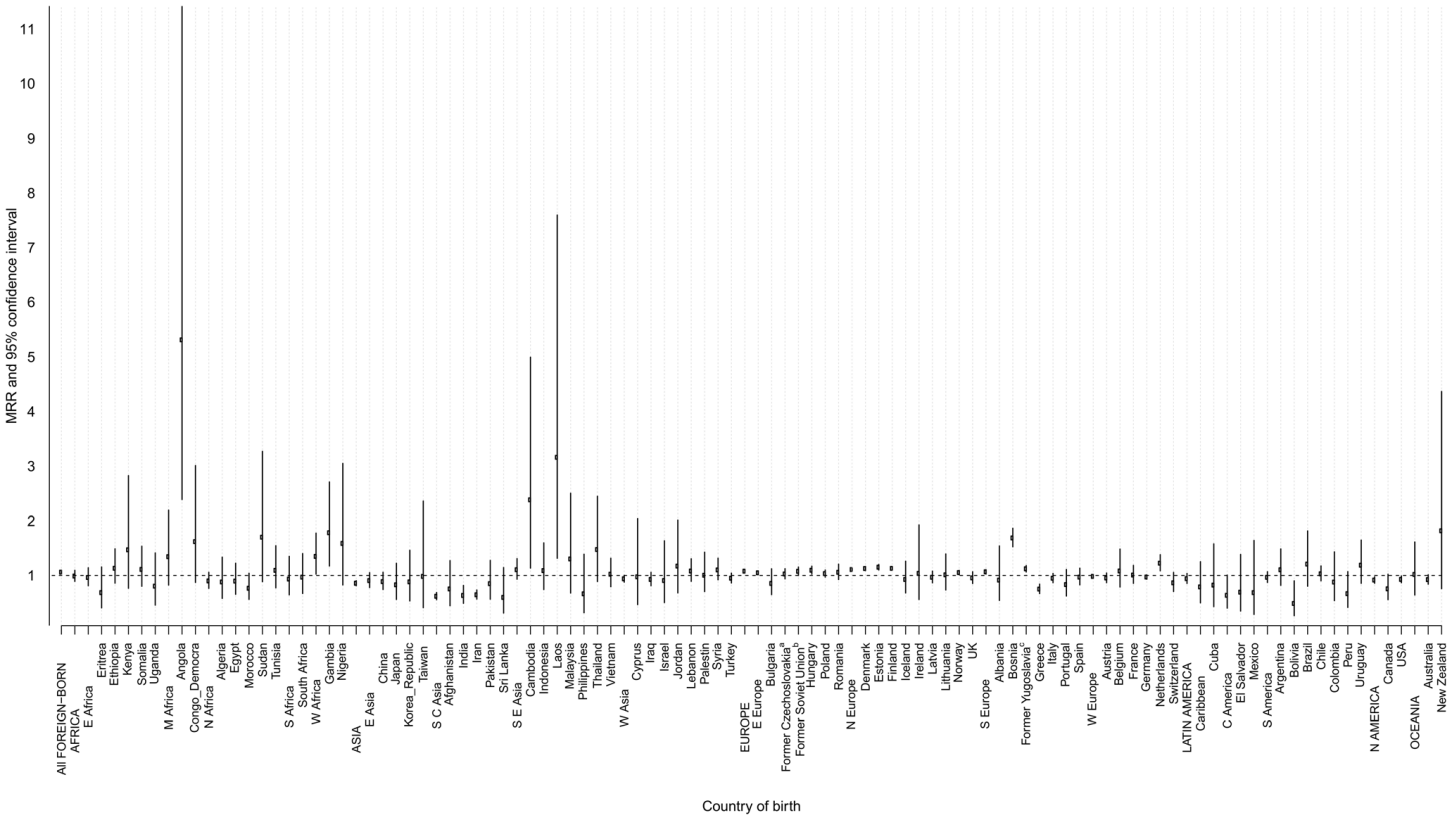
All-site cancer mortality rate ratio (MRR)^*^ and 95% confidence interval (CI) among foreign-born men by continent, region, and country of birth compared with Sweden-born men, 1961–2009. ??? Adjusted for age at follow-up and calendar period at baseline. ^a^The former Czechoslovakia includes Czechoslovakia, Slovakia, and the Czech Republic. ^b^The former Soviet Union includes Belarus, Moldova, Russian Federation, Soviet Union, and Ukraine. ^c^The former Yugoslavia includes Yugoslavia, Croatia, Macedonia, Serbia, Slovenia, and Montenegro.

**Figure 4:**
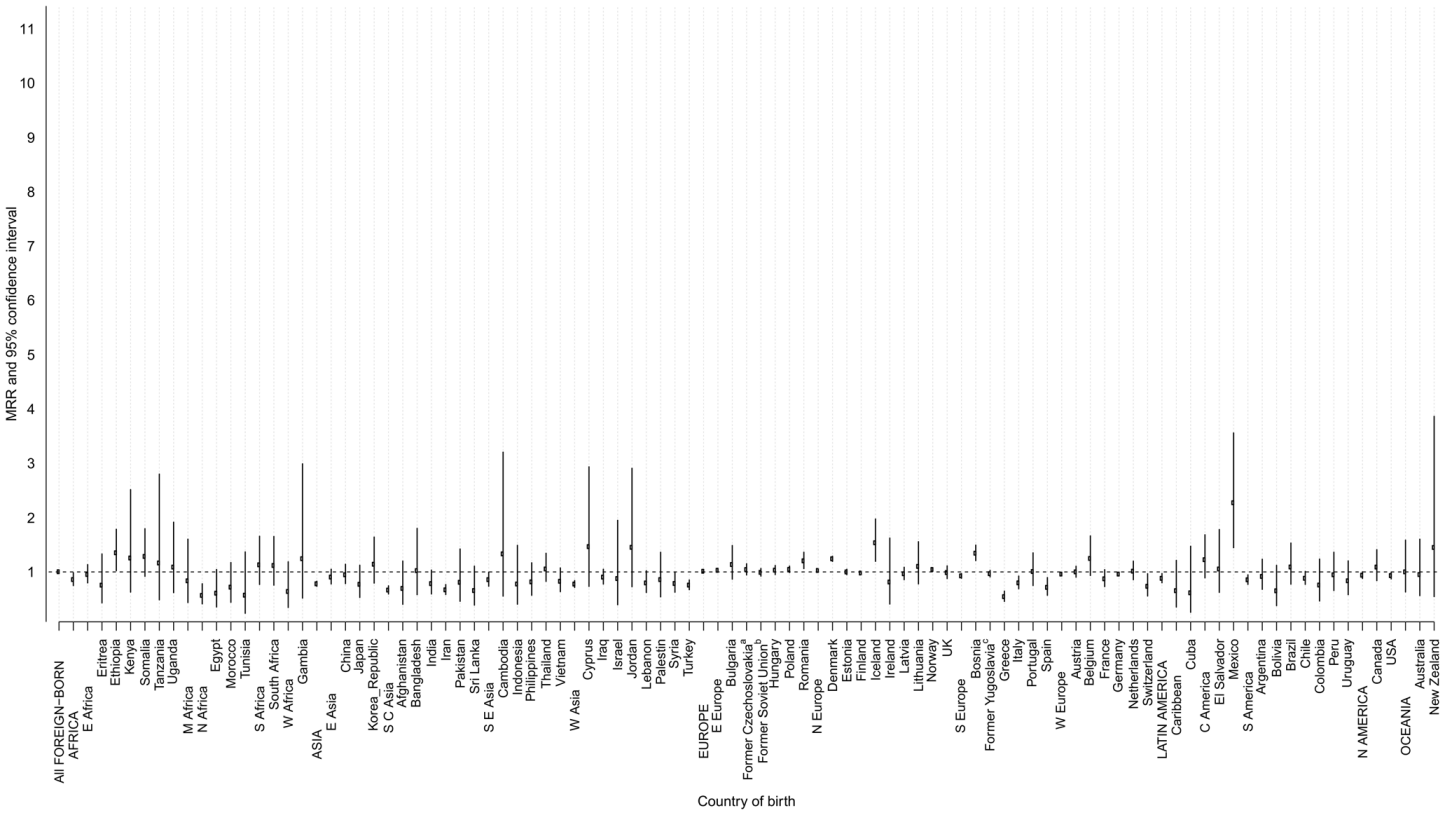
All-site cancer mortality rate ratio (MRR)^*^ and 95% confidence interval (CI) among foreign-born women by continent, region, and country of birth compared with Sweden-born women, 1961–2009. *Adjusted for age at follow-up and calendar period at baseline. ^a^The former Czechoslovakia includes Czechoslovakia, Slovakia, and Czech Republic. ^b^The former Soviet Union includes Belarus, Moldova, Russian Federation, Soviet Union, and Ukraine. ^c^The former Yugoslavia includes Yugoslavia, Croatia, Macedonia, Serbia, Slovenia, and Montenegro.

Foreign-born men had an overall 5% increased all-site cancer mortality risk compared with Sweden-born men after adjustment for age and calendar period at baseline (MRR = 1.05, 95% CI 1.04–1.07) (Figure 3 and Table S1), whereas foreign-born and Sweden-born women had similar risks (MRR = 1.0, 95% CI 0.98–1.01) (Figure 4 and Table S2). Age-specific analysis revealed a lower cancer mortality risk among foreign-born women in all age strata whereas the increased mortality among foreign-born men was limited to those aged between 50 and 70 years (Table 4).

**Table 4:** Cancer mortality rate ratio (MRR) and 95% confidence interval (CI) in foreign-born compared with Sweden-born men and women by cancer type, sex, and age at follow-up in Sweden, 1961–2009.

Age at follow-up**	All-site cancers	Colon cancer	Lung cancer	Stomach cancer	Prostate cancer
Men	MRR* (95% CI)	MRR* (95% CI)	MRR* (95% CI)	MRR* (95% CI)	MRR* (95% CI)
0–9	**0.24 (0.17–0.35)**	-	**-**	**-**	**-**
10–19	**0.24 (0.19–0.30)**	-	**-**	**-**	**-**
20–29	**0.15 (0.12 0.17)**	**0.12 (0.04–0.30)**	**0.45 (0.22–0.88)**	0.55 (0.24–1.23)	-
30–39	**0.44 (0.40–0.49)**	**0.34 (0.23–0.52)**	**0.58 (0.42–0.82)**	0.89 (0.64–1.24)	**-**
40–49	**0.75 (0.71–0.79)**	**0.47 (0.37–0.59)**	**1.26 (1.13–1.40)**	**0.78 (0.65–0.93)**	0.58 (0.33–1.01)
50–59	**1.07 (1.03–1.10)**	**0.82 (0.72–0.93)**	**1.74 (1.64–1.83)**	**1.14 (1.02–1.27)**	**0.74 (0.63–0.86)**
60–69	**1.10 (1.08–1.13)**	0.94 (0.86–1.03)	**1.71 (1.64–1.78)**	**1.29 (1.19–1.40)**	**0.65 (0.60–0.70)**
70–79	0.99 (0.97–1.02)	**0.88 (0.81–0.96)**	**1.58 (1.52–1.65)**	**1.19 (1.10–1.29)**	**0.66 (0.62–0.70)**
80+	**0.79 (0.76–0.81)**	**0.81 (0.73–0.90)**	**1.36 (1.26–1.47)**	0.99 (0.88–1.10)	**0.58 (0.55–0.62)**

	All-site cancers	Colon cancer	Lung cancer	Stomach cancer	Breast cancer	Cervical cancer
Women	MRR* (95% CI)	MRR* (95% CI)	MRR* (95% CI)	MRR* (95% CI)	MRR* (95% CI)	MRR* (95% CI)
0–9	**0.30 (0.22–0.42)**	-	**-**	**-**	**-**	**-**
10–19	**0.22 (0.21–0.28)**	-	**-**	**-**	**-**	**-**
20–29	**0.21 (0.18 0.25)**	**0.34 (0.16 0.72)**	0.45 (0.20–1.03)	0.58 (0.29–1.15)	**0.51 (0.30–0.86)**	**-**
30–39	**0.41 (0.38–0.45)**	**0.26 (0.17–0.41)**	**0.54 (0.37–0.79)**	0.90 (0.66–1.24)	**0.49 (0.41–0.57)**	**0.30 (0.23–0.38)**
40–49	**0.63 (0.60–0.66)**	**0.57 (0.46–0.70)**	**0.82 (0.72–0.95)**	1.04 (0.87–1.25)	**0.68 (0.62–0.74)**	**0.57 (0.48–0.68)**
50–59	**0.79 (0.77–0.81)**	**0.73 (0.64–0.82)**	**0.80 (0.74–0.87)**	**1.18 (1.03–1.35)**	**0.79 (0.74–0.84)**	1.00 (0.86–1.17)
60–69	**0.92 (0.90–0.94)**	**0.84 (0.76–0.92)**	1.02 (0.96–1.09)	**1.23 (1.10–1.36)**	**0.87 (0.82–0.93)**	**1.37 (1.19–1.58)**
70–79	**0.95 (0.93–0.97)**	**0.90 (0.83–0.96)**	**1.24 (1.17–1.32)**	**1.14 (1.04–1.24)**	**0.84 (0.78–0.89)**	**1.27 (1.08–1.49)**
80+	**0.88 (0.86–0.90)**	**0.86 (0.79–0.93)**	**1.29 (1.18–1.41)**	1.00 (0.90–1.11)	**0.77 (0.71–0.83)**	1.09 (0.87–1.37)

*Mortality rate ratios (MRRs) are adjusted for calendar period of follow-up and level of education. The reference group is Sweden-born individuals. MRR values significantly different from 1.0 are highlighted in bold.

**Categories with at least five cases of cancer mortality.

Compared with Sweden-born men, all-site cancer mortality was higher in men born in other European countries (MRR = 1.07, 95% CI 1.06–1.09) (Figure 3 and Table S1). Among immigrant men from Europe, the highest MRR was observed among those born in Bosnia (MRR = 1.68, 95% CI 1.52–1.86) followed by men born in the Netherlands, Estonia, Denmark, Finland, the former Yugoslavia, and Hungary with around a 10% increased risk, and those born in Norway had only a 5% increased risk (MRR = 1.05, 95% CI 1.01–1.09). Men born in other parts of Europe had similar MRRs to those born in Sweden, except for men born in Greece who had a 26% reduced risk (MRR = 0.74, 95% CI 0.66–0.84) (Figure 3 and Table S1). By contrast, men born in Asia and North America had 15% and 10%, respectively, lower all-site cancer mortality compared with men born in Sweden (Figure 3 and Table S1). Men born in Iran and India had the lowest MRRs with about 35% statistically significant reduced risk. The only immigrant group from these continents who had an increased MRR were men born in Laos (MRR = 3.15, 95% CI 1.31–7.59) and Cambodia (MRR = 2.37, 95% CI 1.13–4.99). Overall, men born in Africa had a similar MRR to Sweden-born men whereas African men born in Angola had a five-fold increased risk (MRR = 5.31, 95% CI 2.38–11.8) (Figure 3 and Table S1).

Women born in all parts of the world had lower or similar MRRs compared to women born in Sweden (Figure 4 and Table S2) except for those born in Mexico, Iceland, Denmark, Romania, and Norway in whom the MRR was elevated. The lowest mortality risk (MRR between 0.54 and 0.78) was observed among women born in Greece, Iran, Spain, Switzerland, Turkey, and Syria (Figure 4 and Table S2).

### Specific cancer types

#### Colon cancer

Compared with Sweden-born men and women, the crude MR for colon cancer was lower in foreign-born men (11.2 versus 17.9 per 100,000 person-years) and women (12.9 versus 18.9 per 100,000 person-years) (Table 2), and in all age strata in the age-specific MRR analysis (Table 4). Furthermore, compared with Sweden-born individuals, immigrants had a 9% lower risk of colon cancer mortality after multivariable adjustment in both sexes (men: MRR = 0.91, 95% CI 0.86–0.96; women: MRR = 0.91, 95% CI 0.87–0.95). At the country level, a decreased or similar risk of colon cancer mortality was found among men and women born in all studied countries except men born in Latvia, in whom the risk was elevated by 40%, and women born in Denmark, who had a 30% elevated risk (Tables 5 and 6).

**Table 5:** Cancer mortality rate ratio (MRR) and 95% confidence interval (CI) in men by continent, region, and country of birth and cancer type in Sweden, 1961–2009.

Country of birth**	Colon cancer	Lung cancer	Stomach cancer	Prostate cancer
	MRR* (95% CI)	MRR* (95% CI)	MRR* (95% CI)	MRR* (95% CI)
**Sweden**	1	1	1	1
**Outside Sweden**	**0.91 (0.86–0.96)**	**1.65 (1.60–1.69)**	0.99 (0.95–1.04)	**0.72 (0.69–0.74)**
**Africa**	0.70 (0.45–1.11)	1.07 (0.86–1.33)	0.76 (0.48–1.18)	0.74 (0.49–1.09)
**Eastern Africa**	**-**	0.75 (0.49–1.15)	0.57 (0.23–1.38)	0.72 (0.36–1.46)
Ethiopia	-	0.95 (0.49–1.84)	**-**	**-**
Somalia	**-**	0.86 (0.38–1.94)	**-**	**-**
**North Africa**	0.59 (0.28–1.24)	1.17 (0.85–1.61)	0.80 (0.41–1.56)	**0.42 (0.19–0.88)**
Algeria	**-**	0.98 (0.40–2.36)	**-**	**-**
Egypt	**-**	1.30 (0.71–2.35)	**-**	**-**
Morocco	**-**	1.02 (0.56–1.85)	**-**	**-**
Tunisia	**-**	1.49 (0.77–2.87)	**-**	**-**
**Southern Africa**	**-**	1.34 (0.64–2.81)	**-**	1.00 (0.41–2.42)
South Africa	**-**	1.40 (0.66–2.94)	**-**	1.02 (0.42–2.46)
**Western Africa**	**-**	1.64 (0.95–2.84)	**-**	**-**
Gambia	**-**	1.86 (0.77–4.48)	**-**	**-**
**Asia**	**0.59 (0.46–0.75)**	1.01 (0.91–1.13)	0.89 (0.72–1.10)	**0.39 (0.31–0.49)**
**Eastern Asia**	0.84 (0.47–1.50)	0.71 (0.48–1.05)	1.06 (0.62–1.81)	**0.52 (0.32–0.84)**
China	0.82 (0.42–1.59)	0.77 (0.50–1.18)	0.92 (0.48–1.79)	**0.44 (0.25–0.79)**
**South_Central Asia**	**0.59 (0.39–0.90)**	**0.47 (0.36–0.61)**	**0.55 (0.35–0.86)**	**0.42 (0.29–0.62)**
India	-	0.62 (0.34–1.12)	-	-
Iran	0.64 (0.40–1.04)	**0.45 (0.33–0.62)**	0.72 (0.43–1.21)	**0.49 (0.31–0.76)**
South_Eastern Asia	0.82 (0.46–1.48)	1.17 (0.81.1.68)	0.77 (0.34–1.73)	**-**
Indonesia	**-**	1.57 (0.75–3.31)	**-**	**-**
Vietnam	**-**	1.03 (0.61–1.76)	**-**	**-**
**Western Asia**	**0.56 (0.40–0.78)**	**1.35 (1.19–1.54)**	1.12 (0.87–1.43)	**0.28 (0.20–0.39)**
Iraq	0.64 (0.36–1.15)	1.13 (0.87–1.46)	1.20 (0.74–1.96)	**0.30 (0.15–0.60)**
Lebanon	**-**	1.41 (0.99–2.02)	1.03 (0.45–2.33)	**-**
Palestinian territories	**-**	1.36 (0.73–2.54)	**-**	**-**
Syrian Arab Republic	**-**	**1.75 (1.30–2.36)**	1.16 (0.57–2.37)	**0.34 (0.14–0.83)**
Turkey	**0.51 (0.32–0.82)**	**1.51 (1.27–1.80)**	1.34 (0.97–1.86)	**0.23 (0.15–0.37)**
**Europe**	**0.92 (0.87–0.97)**	**1.70 (1.65–1.75)**	1.01 (0.97–1.06)	**0.72 (0.69–0.75)**
**Eastern Europe**	1.02 (0.89–1.16)	**1.55 (1.44–1.66)**	**1.18 (1.05–1.33)**	**0.60 (0.54–0.67)**
Bulgaria	**-**	1.11 (0.64–1.93)	1.19 (0.49–2.88)	**-**
Former Czechoslovakia^a^	1.03 (0.75–1.43)	**1.55 (1.29–1.85)**	1.12 (0.82–1.52)	**0.66 (0.50–0.86)**
Former Soviet Union^b^	1.00 (0.76–1.31)	**1.57 (1.35–1.82)**	**1.53 (1.24–1.88)**	**0.66 (0.54–0.81)**
Hungary	1.03 (0.80–1.33)	**1.83 (1.62–2.08)**	0.93 (0.72–1.20)	**0.63 (0.51–0.79)**
Poland	1.12 (0.89–1.39)	**1.36 (1.19–1.55)**	**1.25 (1.01–1.54)**	**0.53 (0.43–0.65)**
Romania	0.51 (0.26–1.00)	**1.64 (1.29–2.08)**	0.95 (0.55–1.62)	**0.51 (0.32–0.82)**
**Northern Europe**	**0.92 (0.87–0.98)**	**1.78 (1.72–1.83)**	1.03 (0.97–1.09)	**0.77 (0.74–0.81)**
Denmark	0.95 (0.83–1.08)	**1.93 (1.81–2.06)**	**0.66 (0.56–0.77)**	**0.72 (0.65–0.80)**
Estonia	1.12 (0.96–1.32)	**1.51 (1.37–1.66)**	**1.55 (1.36–1.77)**	**0.82 (0.73–0.92)**
Finland	**0.77 (0.69–0.85)**	**1.99 (1.91–2.08)**	1.07 (0.99–1.17)	**0.75 (0.69–0.80)**
Iceland	**-**	**1.92 (1.17–3.15)**	**-**	**-**
Latvia	**1.40 (1.00–1.95)**	1.03 (0.78–1.36)	1.11 (0.78–1.57)	0.86 (0.67–1.12)
Lithuania	**-**	**1.88 (1.04–3.40)**	**-**	0.80 (0.36–1.79)
Norway	1.08 (0.94–1.24)	**1.36 (1.25–1.48)**	1.01 (0.88–1.16)	**0.84 (0.76–0.93)**
UK	0.98 (0.66–1.47)	1.07 (0.84–1.38)	0.92 (0.60–1.40)	**0.67 (0.47–0.94)**
**Southern Europe**	**0.76 (0.64–0.90)**	**1.84 (1.71–1.97)**	**1.15 (1.01–1.32)**	**0.39 (0.33–0.46)**
Bosnia	1.18 (0.75–1.85)	**2.64 (2.20–3.15)**	**2.08 (1.41–3.07)**	**0.54 (0.33–0.87)**
Former Yugoslavia^c^	**0.70 (0.55–0.89)**	**1.95 (1.78–2.15)**	**1.36 (1.13–1.62)**	**0.41 (0.32–0.52)**
Greece	0.73 (0.47–1.12)	1.23 (0.98–1.50)	0.80 (0.53–1.21)	**0.22 (0.12–0.41)**
Italy	0.78 (0.55–1.12)	1.93 (1.65–2.26)	0.79 (0.56–1.12)	**0.34 (0.24–0.48)**
Portugal	**-**	0.81 (0.42–1.56)	**-**	**-**
Spain	0.58 (0.27–1.22)	**1.49 (1.10–2.00)**	0.63 (0.31–1.26)	**0.45 (0.24–0.85)**
**Western Europe**	0.92 (0.81–1.05)	**1.41 (1.31–1.51)**	**0.75 (0.66–0.86)**	**0.75 (0.68–0.82)**
Austria	0.83 (0.57–1.19)	**1.45 (1.20–1.75)**	1.00 (0.73–1.37)	**0.54 (0.40–0.73)**
Belgium	**-**	**1.85 (1.02–3.34)**	-	0.94 (0.42–2.10)
France	1.37 (0.82–2.27)	0.96 (0.64–1.45)	0.60 (0.28–1.26)	0.92 (0.61–1.39)
Germany	0.95 (0.82–1.11)	**1.34 (1.23–1.46)**	**0.78 (0.67–0.92)**	**0.75 (0.67–0.84)**
Netherlands	0.72 (0.41–1.27)	**2.44 (1.99–3.01)**	**0.38 (0.18–0.81)**	0.87 (0.61–1.22)
Switzerland	0.71 (0.32–1.60)	1.30 (0.87–1.95)	**-**	1.03 (0.66–1.60)
**Latin America**	0.69 (0.45–1.07)	0.95 (0.77–1.18)	**1.53 (1.12–2.08)**	0.88 (0.65–1.19)
**Caribbean**	**-**	**-**	**-**	1.78 (0.74–4.29)
**Central America**	**-**	0.88 (0.36–2.12)	**-**	**-**
**South America**	0.70 (0.45–1.10)	0.98 (0.78–1.22)	**1.67 (1.21–2.31)**	0.73 (0.53–1.03)
Argentina	**-**	1.20 (0.62–2.31)	**-**	**-**
Chile	0.56 (0.29–1.06)	0.95 (0.71–1.27)	**2.54 (1.75–3.70)**	0.78 (0.51–1.20)
Colombia	**-**	1.55 (0.64–3.75)	**-**	**-**
Uruguay	**-**	**1.87 (1.08–3.24)**	**-**	**-**
**North America**	0.94 (0.74–1.18)	0.99 (0.85–1.16)	**0.59 (0.45–0.79)**	0.90 (0.77–1-04)
Canada	**-**	0.53 (0.22–1.28)	**-**	0.89 (0.44–1.78)
USA	0.95 (0.75–1.21)	1.03 (0.87–1.21)	**0.61 (0.46–0.81)**	0.89 (0.77–1.04)

*Mortality rate ratios (MRRs) are adjusted for age at follow-up and calendar period at baseline. The reference group is Sweden-born men. MRR values significantly different from 1.0 are highlighted in bold.

**Continents, regions, and countries with at least five cases of cancer mortality.

a The former Czechoslovakia includes Czechoslovakia, Slovakia, and the Czech Republic.

b The former Soviet Union includes Belarus, Moldova, Russian Federation, Soviet Union, and Ukraine.

c The former Yugoslavia includes Yugoslavia, Croatia, Macedonia, Serbia, Slovenia, and Montenegro.

**Table 6:** Cancer mortality rate ratio (MRR) and 95% confidence interval (CI) in women by continent, region, and country of birth and cancer type in Sweden, 1961–2009.

Country of birth**	Colon cancer	Lung cancer	Stomach cancer	Breast cancer	Cervical cancer
	MRR* (95% CI)	MRR* (95% CI)	MRR* (95% CI)	MRR* (95% CI)	MRR* (95% CI)
**Sweden**	1	1	1	1	1
**Outside Sweden**	**0.91 (0.87–0.95)**	**1.34 (1.29–1.40)**	1.03 (0.98–1.09)	**0.92 (0.89–0.95)**	**1.22 (1.14–1.32)**
**Africa**	**0.49 (0.25–0.96)**	0.71 (0.54–1.12)	0.49 (0.21–1.10)	0.97 (0.72–1.30)	0.53 (0.21–1.29)
**Eastern Africa**	**-**	0.67 (0.36–1.22)	**-**	1.11 (0.77–1.61)	**-**
Eritrea	**-**	**-**	**-**	1.42 (0.58–3.45)	**-**
Ethiopia	**-**	**-**	**-**	1.50 (0.82–2.73)	**-**
Somalia	**-**	**-**	**-**	0.71 (0.31–1.61)	**-**
**North Africa**	**-**	**-**	**-**	0.78 (0.42–1.46)	**-**
Morocco	**-**	**-**	**-**	1.09 (0.45–2.63)	**-**
**Southern Africa**	**-**	**-**	**-**	1.50 (0.67–1.34)	**-**
South Africa	**-**	**-**	**-**	1.56 (0.70–3.48)	**-**
**Asia**	**0.61 (0.48–0.78)**	**0.77 (0.64–0.92)**	**1.26 (1.00–1.58)**	**0.77 (0.66–0.89)**	**0.68 (0.47–0.98)**
**Eastern Asia**	1.01 (0.61–1.66)	1.25 (0.85–1.84)	**1.72 (1.05–2.80)**	**0.58 (0.37–0.90)**	1.04 (0.46–2.34)
China	0.82 (0.42–1.59)	**1.57 (1.01–2.43)**	1.06 (0.50–2.25)	0.64 (0.37–1.11)	**-**
Korean_Republic	**-**	**-**	**6.16 (2.75–13.8)**	**-**	**-**
**South_Central Asia**	**0.37 (0.22–0.62)**	**0.46 (0.32–0.67)**	0.90 (0.57–1.42)	0.84 (0.65–1.07)	0.51 (0.25–1.03)
India	0.95 (0.39–2.29)	**-**	**-**	0.78 (0.40–1.51)	**2.49 (1.03–6.05)**
Iran	**0.24 (0.11–0.50)**	**0.50 (0.32–0.78)**	1.04 (0.61–1.76)	0.94 (0.71–1.26)	**-**
**South_Eastern Asia**	0.82 (0.46–1.48)	0.83 (0.54–1.29)	**1.85 (1.12–3.06)**	0.72 (0.50–1.03)	1.46 (0.81–2.61)
Philippines	**-**	**-**	**-**	0.72 (0.32–1.62)	**-**
Thailand	**-**	1.40 (0.75–2.64)	**2.72 (1.27–5.86)**	0.97 (0.58–1.63)	**-**
Vietnam	0.83 (0.34–2.03)	0.57 (0.23–1.39)	**2.73 (1.38–5.38)**	0.45 (0.20–1.01)	**3.10 (1.43–6.71)**
**Western Asia**	**0.56 (0.40–0.78)**	**0.71 (0.55–0.90)**	1.29 (0.95–1.75)	**0.79 (0.65–0.96)**	**0.37 (0.19–0.72)**
Iraq	1.04 (0.61–1.76)	0.61 (0.37–1.01)	0.74 (0.35–1.54)	**1.34 (1.00–1.81)**	**-**
Lebanon	0.75 (0.30–1.84)	1.11 (0.60–2.03)	**-**	0.91 (0.53–1.55)	**-**
Syrian Arab Republic	**-**	**0.39 (0.16–0.95)**	**1.99 (1.00–3.93)**	0.97 (0.60–1.55)	**-**
Turkey	**0.37 (0.22–0.64)**	0.79 (0.57–1.11)	**1.75 (1.21–2.52)**	**0.44 (0.30–0.63)**	0.63 (0.28–1.44)
**Europe**	**0.91 (0.87–0.96)**	**1.37 (1.32–1.43)**	1.05 (0.99–1.10)	**0.92 (0.88–0.95)**	**1.24 (1.15–1.34)**
**Eastern Europe**	1.01 (0.89–1.14)	**1.24 (1.12–1.38)**	1.12 (0.97–1.30)	0.94 (0.83–1.03)	1.18 (0.95–1.47)
Bulgaria	1.14 (0.47–2.77)	1.07 (0.48–2.40)	**-**	1.17 (0.63–2.19)	**-**
Former Czechoslovakia^a^	1.02 (0.73–1.42)	1.07 (0.79–1.46)	0.86 (0.55–1.36)	1.06 (0.83–1.35)	**1.84 (1.12–3.01)**
Former Soviet Union^b^	1.16 (0.94–1.44)	0.82 (0.63–1.06)	**1.48 (1.17–1.87)**	**0.74 (0.60–0.91)**	0.88 (0.53–1.48)
Hungary	1.05 (0.80–1.37)	**1.63 (1.33–2.01)**	0.85 (0.58–1.23)	1.02 (0.84–1.24)	1.04 (0.62–1.73)
Poland	0.91 (0.74–1.12)	**1.38 (1.18–1.61)**	1.05 (0.82–1.35)	0.95 (0.83–1.10)	1.05 (0.73–1.52)
Romania	0.72 (0.43–1.22)	**1.54 (1.11–2.13)**	**1.65 (1.04–2.63)**	1.20 (0.88–1.63)	**2.17 (1.20–3.92)**
**Northern Europe**	**0.91 (0.86–0.96)**	**1.46 (1.40–1.52)**	**1.06 (1.00–1.13)**	**0.90 (0.86–0.94)**	**1.31 (1.20–1.43)**
Denmark	**1.29 (1.13–1.46)**	**2.46 (2.24–2.69)**	**0.81 (0.66–0.98)**	1.03 (0.93–1.14)	**1.92 (1.58–2.35)**
Estonia	1.13 (0.97–1.32)	0.97 (0.81–1.15)	**1.54 (1.31–1.81)**	**0.79 (0.68–0.92)**	1.34 (0.99–1.82)
Finland	**0.71 (0.65–0.77)**	**1.31 (1.24–1.39)**	**1.11 (1.02–1.20)**	**0.89 (0.84–0.93)**	1.00 (0.88–1.14)
Iceland	**-**	**3.31 (1.95–5.60)**	**-**	1.57 (0.89–2.78)	**-**
Latvia	1.21 (0.86–1.70)	1.21 (0.84–1.75)	1.12 (0.73–1.73)	0.79 (0.57–1.11)	**1.93 (1.07–3.50)**
Norway	1.01 (0.91–1.13)	**1.51 (1.38–1.65)**	0.95 (0.83–1.09)	**0.88 (0.82–0.97)**	**1.89 (1.61–2.22)**
UK	1.32 (0.94–1.87)	1.25 (0.88–1.76)	0.54 (0.28–1.05)	1.09 (0.83–1.43)	0.85 (0.38–1.90)
**Southern Europe**	**0.67 (0.55–0.82)**	1.05 (0.91–1.21)	1.17 (0.96–1.42)	0.90 (0.80–1.01)	1.04 (0.79–1.37)
Bosnia	0.96 (0.62–1.47)	**1.65 (1.24–2.19)**	**1.68 (1.07–2.63)**	0.99 (0.75–1.31)	1.25 (0.67–2.34)
Former Yugoslavia^c^	**0.66 (0.51–0.86)**	1.06 (0.88–1.26)	1.25 (0.96–1.62)	0.99 (0.86–1.15)	1.27 (0.92–1.77)
Greece	**0.46 (0.24–0.89)**	**0.30 (0.15–0.61)**	0.69 (0.41–1.53)	0.76 (0.54–1.06)	**-**
Italy	0.64 (0.37–1.10)	1.29 (0.88–1.90)	0.88 (0.50–1.56)	0.69 (0.47–1.02)	**-**
Portugal	**-**	**-**	**-**	1.19 (0.64–2.22)	**-**
Spain	**-**	0.94 (0.50–1.76)	1.05 (0.47–2.34)	0.74 (0.44–1.25)	**-**
**Western Europe**	0.93 (0.83–1.04)	**1.13 (1.02–1.25)**	0.88 (0.77–1.02)	0.99 (0.91–1.07)	1.02 (0.83–1.26)
Austria	0.73 (0.49–1.09)	1.06 (0.76–1.47)	1.03 (0.69–1.54)	1.17 (0.93–1.48)	1.09 (0.58–2.03)
Belgium	**-**	**-**	**-**	**2.10 (1.22–3.63)**	**-**
France	0.79 (0.42–1.47)	0.79 (0.42–1.47)	0.70 (0.31–1.57)	1.09 (0.73–1.61)	1.46 (0.60–3.52)
Germany	0.97 (0.86–1.10)	**1.15 (1.03–1.29)**	0.93 (0.80–1.08)	0.94 (086–1.04)	0.99 (0.78–1.25)
Netherlands	1.01 (0.57–1.79)	1.41 (0.89–2.25)	**-**	1.81 (0.81–1.72)	1.71 (0.76–3.82)
Switzerland	**-**	0.93 (0.41–2.07)	**-**	0.95 (0.52–1.71)	**-**
**Latin America**	0.61 (0.46–1.00)	1.03 (0.79–1.34)	1.04 (0.70–1.56)	**0.76 (0.59–0.96)**	1.14 (0.69–1.89)
**Central America**	**-**	1.30 (0.54–3.15)	**-**	1.15 (0.54–2.44)	**-**
Mexico	**-**	**-**	**-**	**2.91 (1.21–7.01)**	**-**
**South America**	0.66 (0.44–1.00)	0.92 (0.69–1.22)	1.00 (0.65–1.55)	**0.72 (0.56–0.94)**	1.01 (0.58–1.76)
Argentina	**-**	0.96 (0-40–2.32)	**-**	1.06 (0.55–2.04)	**-**
Bolivia	**-**	-	**-**	1.37 (0.56–3.30)	**-**
Brazil	**-**	**3.37 (1.86–6.10)**	**-**	1.46 (0.76–2.82)	**-**
Chile	0.82 (0.42–1.59)	0.95 (0.66–1.37)	1.30 (0.77–2.19)	**0.53 (0.36–0.80)**	1.08 (0.52–2.24)
Uruguay	**-**	**-**	**-**	1.19 (0.59–2.39)	**-**
**North America**	1.00 (0.81–1.23)	0.94 (0.76–1.17)	0.75 (0.56–1.00)	**1.21 (1.05–1.40)**	1.01 (0.66–1.55)
Canada	**-**	1.42 (0.67–2.98)	**-**	**1.90 (1.18–3.06)**	**-**
USA	1.00 (0.81–1.24)	0.91 (0.72–1.15)	0.76 (0.56–1.02)	**1.17 (1.00–1.37)**	1.04 (0.67–1.61)

*Mortality rate ratios (MRRs) are adjusted for age at follow-up and calendar period at baseline. The reference group is Sweden-born women.

**Continents, regions, and countries with at least five cases of cancer mortality.

a The former Czechoslovakia includes Czechoslovakia, Slovakia, and the Czech Republic.

b The former Soviet Union includes Belarus, Moldova, Russian Federation, Soviet Union, and Ukraine.

c The former Yugoslavia includes Yugoslavia, Croatia, Macedonia, Serbia, Slovenia, and Montenegro.

#### Lung cancer

The crude MR for lung cancer was lower in women than in men in both the Sweden-born and foreign-born cohorts (Table 2). Compared with Sweden-born men and women, the MR was higher in foreign-born men (47.2 versus 36.5 per 100,000 person-years) and women (21.1 versus 17.9 per 100,000 person-years) (Table 2). Age-specific MRR analysis revealed that these increases in risk among foreign-born individuals were confined to men older than 40 years and women older than 70 years of age (Table 4). The risk remained higher in foreign-born individuals even after multivariable adjustment for education level, age, and calendar period at baseline (Tables 5 and 6). In the analysis by specific birth country, we observed increased or similar lung cancer mortality among men born in all studied countries of birth except for those born in Iran, in whom MRR was reduced by 55% (Table 5). Compared with women born in Sweden, lung cancer mortality was higher in women born in almost all countries in Europe but similar or lower in women born in African, Asian and Latin American countries (Table 6).

#### Stomach cancer

The crude MR for stomach cancer was lower in women than in men regardless of country of birth. Mortality was lower in foreign-born men and women younger than 50 years of age, but higher among those 50 years of age and older, compared with Sweden-born men and women, respectively (Tables 2 and 4).

Overall foreign-born men and women had similar risks of stomach cancer mortality compared with Sweden-born men and women, respectively after multivariable adjustment for education level, age, and calendar period at baseline (Tables 5 and 6). At the country level, stomach cancer mortality was higher in men born in Chile, Bosnia, Estonia, the former Soviet Union, the former Yugoslavia, and Poland and lower or similar in men born in the remaining countries studied, compared with Sweden-born men (Table 5). Women in most part of Asia and Northern Europe had higher risks whereas women born in other countries worldwide had similar stomach cancer mortality compared with Sweden-born women (Table 6).

#### Prostate cancer

Foreign-born men had lower MRs and MRRs for prostate cancer and in all age groups and in almost all countries, compared with men born in Sweden (Tables 2, 4 and 5). Foreign-born men had an overall 30% lower risk of prostate cancer mortality compared with Sweden-born men after adjustment for education level, age, and calendar period at baseline (MRR = 0.72, 95% CI 0.69–0.74).

#### Breast cancer

The same lower pattern in foreign-born compared with Sweden-born individuals as for prostate cancer mortality was observed for breast cancer mortality (Tables 2, 4 and 6). Compared with Sweden-born women, foreign-born women had an overall 8% lower risk of breast cancer mortality after adjustment for level of education, age, and calendar period at baseline (MRR = 0.92, 95% CI 0.89–0.95). This reduction in risk was noted for all age groups (Table 4). A reduction in risk was also observed among foreign-born women from all countries except Mexico, Belgium, Canada, Iraq, and the USA, in whom breast cancer MRR values were increased (Table 6).

#### Cervical cancer

In contrast to breast cancer, the cervical cancer MR and MRR were both higher in foreign-born women compared to those born in Sweden (Tables 2 and 6), but only among women older than 60 years of age (Table 4). In the country-specific analysis, we observed higher cervical cancer mortality in women born in almost all countries studied with the highest mortality in those born in Vietnam, followed by India, Romania, Latvia, Denmark, Norway, and the former Czechoslovakia (Table 6).

## Discussion

In this nationwide cohort study we observed an overall downward trend in all-site cancer mortality among men over the past three decades but no change among women over the past two decades, regardless of country of birth. We further found a consistently higher risk of all-site cancer mortality among individuals with a low level of education compared with those who were more highly educated regardless of sex and country birth over the entire study period. All-site cancer mortality was overall slightly increased among foreign-born men compared with Sweden-born men, whereas foreign-born women had a similar risk for all-site cancer mortality compared with Sweden-born women. The results varied for site-specific cancers. Foreign-born individuals had lower risks of colon, prostate, and breast cancer mortality but higher lung and cervical cancer mortality compared with the Sweden-born group.

The present study is based on the total population of Sweden with a mixed group of foreign-born individuals and complete information on country of birth. We had complete information from follow-up during the past five decades through linkage between several unique and high-quality Swedish registers. The quality of the data in the Cause of Death Register varies, mainly according to the quality of the assessment of the cause of death and the accuracy with which the physician has filled out the death certificate. However, it has been shown that the underlying cause of death for malignant neoplasms is up to 90% reliable [18].

The observed increased risk of all-site cancer mortality among individuals of either sex with a low level of education during all study periods, except the years 1961–1970, is in line with the results of studies conducted in Australia and Spain [19], [20]. This finding could be due in part to death from cancers with a poor prognosis and a higher prevalence among individuals with a low SEP, such as stomach cancer [19], [21], different health-seeking behavior leading to late-stage cancer at diagnosis, or less favorable lifestyles [22] and related comorbidities, leading to increased mortality in this group. The observed reverse association between mortality and education (i.e. decreased mortality with decreasing level of education during the period 1960–1970) is in line with the results of studies from Barcelona, Spain and from Puerto Rico [23], [24]. However, this finding should be interpreted with caution, as the missing percentage of information on level of education was higher during the first decade of the present study (1960–1970) compared with other periods.

The finding of increased risk of all-site cancer mortality among all foreign-born men is in contrast with the results from some previous studies [7], [8]. However, similar to our findings, higher all-site cancer mortality among men born in West Africa has been reported from the UK [25], [26]. The lower all-site cancer mortality among men born in Asia and in South East Asia in particular, in our study has been reported previously [25], [27], [28]. Our finding of a higher risk among men born in Eastern and Southern Europe is in contrast to results reported from Spain [8], [28], [29].

The similar risk of all-site cancer mortality among all foreign-born women in our study is in line with results from some studies [7], [33], but in contrast to others [8], [29], [30]. Similar lower all-site cancer mortality risks among women born in Turkey, Asia, South East Asia, Italy, East and North Africa, Southern Europe, and in South America have been reported previously [8], [25], [26], [27], [29], [31], [32], [33], [34]. In contrast to our results, all-site cancer mortality was reported to be lower in Mexican women living in the USA than in women born within America [35]. However, the fact that in many cases death certificates in the USA are unavailable, especially in the southern states with a large population of mainly Mexican immigrants [35], might have resulted in decreased mortality in immigrants in the American study.

It has been proposed that a lack of reporting of emigration back to their country of origin among elderly ill foreign-born individuals prior to death may be associated with some of the observed decreased mortality in this group [34], [36], [37]. However, the opposite findings of increased mortality in foreign-born men and similar mortality risk in foreign-born women, compared with Sweden-born individuals, do not support this hypothesis. In addition the results of the sensitivity analysis revealed a very low and similar proportion of Sweden-born and foreign-born individuals who were 100 years of age and older; thus further refuting the hypothesis of non-reported emigration of older foreign-born men and women.

### Specific cancer types

#### Colon cancer

The finding of lower risk of colon cancer mortality among foreign-born men and women from Southern Europe and Turkey is in line with the results of other studies [8]. In contrast to our finding, higher risks of colon cancer mortality were observed among foreign-born men and women from Middle Eastern countries in a study in California [38]. The differences in colon cancer mortality risk might be due to differences in lifestyle-related factors including diet and alcohol consumption which are most likely to play an important role in the reported colon cancer mortality risks [8], [39], [40].

#### Lung cancer

The finding of higher lung cancer mortality in foreign-born men from the former Soviet Union and Southern Europe in our study is in line with the results of previous studies [6], [28]. In contrast to our results, lower risks among foreign-born men from Asia, Western Africa, and Turkey and among foreign-born women from Eastern Europe were observed in other studies [6], [25], [26], [31], [36]. Our finding of a lower risk of lung cancer mortality among foreign-born women from Asia and South America is also in agreement with reported results from the Netherlands [8], [25], [26], [29]. The prevalence of smoking, the causal risk factor for lung cancer [6], [26], [41], and genetic susceptibility [40] are the most probable explanations for the observed risk differences among foreign-born compared with Sweden-born men and women. Unfortunately, reliable data at the national level on smoking habits among foreign-born individuals in Sweden are lacking. However a cross-sectional survey in Stockholm County in 2006 showed a higher prevalence of daily smoking among women born in Eastern Europe, Turkey, Bosnia, and Finland, and a lower prevalence among women born in parts of Southern and Central Africa, compared with Sweden-born women [42].

#### Stomach cancer

Our findings of higher stomach cancer mortality risks in men born in Eastern Europe and South America and women born in most countries in Asia, compared with Sweden-born individuals, are supported by previous observations [6], [27], [31], [33], [40], [43], [44], [45]. The higher risk of stomach cancer mortality among foreign-born individuals could be explained by the following factors, in addition to genetic variation: an increased incidence of this cancer as a result of a higher prevalence of Helicobacter pylori infection; and different dietary habits such as a higher consumption of salty foods [6], .

#### Prostate cancer

The observed lower risk of prostate cancer mortality among foreign-born men, which was also found in immigrants in other parts of the world [6], [8], [25], [39], [40], [46], [47], [48], might be due to a true lower incidence of prostate cancer [26], [49] or to undetected prostate cancer among foreign-born men as a result of a lack of awareness among this group of the prostate-specific antigen test. In contrast to our finding, a higher risk among foreign-born men from South America was observed previously [26], [27].

#### Breast cancer

A lower breast cancer mortality risk among foreign-born women, as observed in our study, has been reported previously including in women born in Chile, Turkey, Eastern Asia, Asia, and South America migrating to other parts of the world [8], [25], [26], [27], [29], [31], [39], [40], [41], [45], [46], [50], [51]. The observed lower breast cancer mortality among foreign-born women compared with those born in Sweden could be explained in part by a lower incidence of breast cancers in this group [52] or by death from other diseases such as cardiovascular disorders [53], [54]. However, a higher breast cancer fatality rate among immigrant women in Sweden over the past decade, with a lower or similar rate in previous years, has been reported [52].

#### Cervical cancer

Cervical cancer mortality in immigrants has rarely been studied. A higher mortality risk due to cancer of the cervix has been observed among foreign-born women in the USA compared with US whites [55]. By contrast, foreign-born women in the Netherlands had a lower mortality risk [8]. Late detection of human papilloma virus infection among foreign-born women [40], [56] and thus late diagnosis of cervical cancer is the most probable cause of the higher cervical cancer mortality among the majority of these women.

Although early life exposures in the country of origin and changes in traditional lifestyle factors play an important role in cancer incidence and mortality, suboptimal communication on the dilemmas of treatment and disparities in the access to care could also explain some observed variations in mortality.

We did not have access to clinical data such as severity of disease, stage of cancer, or medical treatment that may have affected mortality risks. Further, we lacked genetic data and information on environmental factors, such as access to healthcare services, social support, degree of acculturation, dietary habits, alcohol consumption, smoking, and physical activities, in order to explore the underlying reasons for cancer mortality differences by country of birth.

The number of missing values regarding education level might be of concern (about 28% and 32% among foreign-born and Sweden-born individuals, respectively). It is possible that this could have been the cause of the observed highest risk among individuals with a low level of education. Given the high risk of cancer mortality in the unknown education category, the observed high risk in the least well educated individuals would be overestimated if the majority in the unknown group had a high education level, and would be underestimated if the majority belonged to the low education group. Although we believe that the latter is more likely, we cannot rule out the alternative possibility. In addition, we observed a similar pattern of the effect of education level in both foreign-born and Sweden-born individuals in the stratified analysis, indicating that the observed higher mortality in individuals with a low education level is a true association and not biased by the unknown education category. Furthermore, using the education variable in the same data in our previous study, exploring the association between breast cancer incidence and low level of education [52], we have seen assuring results of the inverse association between incidence of breast cancer and education, which has been known for many decades to be a risk factor for breast cancer.

Differences in all-sites cancer mortality between men and women in Sweden are declining as a result of a decreasing trend in mortality over time in men but no change in women. A low level of education remains a risk factor for all-sites cancer mortality independent of sex and immigration status. Further studies should explore the underlying reason(s) for the differences in overall risk and specific cancer mortality among foreign-born and Sweden-born individuals.

## Supporting Information

Table S1 **All-site cancer mortality rate ratios (MRR) and 95% confidence interval (CI) in men by continent, region, and country of birth in Sweden, 1961–2009.** *Mortality rate ratios (MRRs) are adjusted for age at follow-up and calendar period at baseline. The reference group is Sweden-born men. MRR values significantly different from 1.0 are highlighted in bold. **Continents, regions, and countries with at least five cases of cancer mortality. ^a^The former Czechoslovakia includes Czechoslovakia, Slovakia, and the Czech Republic. ^b^The former Soviet Union includes Belarus, Moldova, Russian Federation, Soviet Union, and Ukraine. ^c^The former Yugoslavia includes Yugoslavia, Croatia, Macedonia, Serbia, Slovenia, and Montenegro.
(DOC)

Table S2 **All-site cancer mortality rate ratios (MRR) and 95% confidence interval (CI) in women by continent, region, and country of birth in Sweden, 1961–2009.** *Mortality rate ratios (MRRs) are adjusted for age at follow-up and calendar period at baseline. The reference group is Sweden-born women. MRR values significantly different from 1.0 are highlighted in bold. **Continents, regions, and countries with at least five cases of cancer mortality. ^a^The former Czechoslovakia includes Czechoslovakia, Slovakia, and the Czech Republic. ^b^The former Soviet Union includes Belarus, Moldova, Russian Federation, Soviet Union, and Ukraine. ^c^The former Yugoslavia includes Yugoslavia, Croatia, Macedonia, Serbia, Slovenia, and Montenegro.
(DOC)

## References

[1] Lozano R, Naghavi M, Foreman K, Lim S, Shibuya K, et al (2013) Global and regional mortality from 235 causes of death for 20 age groups in 1990 and 2010: a systematic analysis for the Global Burden of Disease Study 2010. Lancet 380: 2095–2128. 10.1016/S0140-6736(12)61728-0 PMC10790329 23245604

[2] World Health Organization (2014) Cancer. Available: http://www.who.int/mediacentre/factsheets/fs297/en/. Accessed 15 February 2013.

[3] World Health Organization. Regional office for Europe, cancer. Available: http://www.euro.who.int/en/what-we-do/health-topics/diseases-and-conditions/cancer. Accessed 2 February 2011.

[4] Socialstyrelsen (2010) Cause of death in Sweden 2010. Available: http://www.socialstyrelsen.se/lists/artikelkatalog/attachments/18394/2011-7-6.pdf. Accessed 14 October 2013.

[5] Moradi T, Allebeck P, Jacobsson A, Mathers C (2006) The burden of disease in Sweden measured with DALY. Neuropsychiatric diseases and cardiovascular diseases dominate. Lakartidningen 103: 137–141. 16465758

[6] Winkler V, Ott JJ, Holleczek B, Stegmaier C, Becher H (2009) Cancer profile of migrants from the Former Soviet Union in Germany: incidence and mortality. Cancer Causes Control 20: 1873–1879. 19543985 10.1007/s10552-009-9381-4

[7] Singh GK, Siahpush M (2001) All-cause and cause-specific mortality of immigrants and native born in the United States. Am J Public Health 91: 392–399. 11236403 10.2105/ajph.91.3.392 PMC1446566

[8] Stirbu I, Kunst AE, Vlems FA, Visser O, Bos V, et al (2006) Cancer mortality rates among first and second generation migrants in the Netherlands: Convergence toward the rates of the native Dutch population. Int J Cancer 119: 2665–2672. 16929492 10.1002/ijc.22200

[9] United Nations, Department of Economic and Social Affairs, Population Division (2009) Trends in International Migrant Stock: The 2008 Revision. Available: http://esa.un.org/migration. Accessed 14 October 2013.

[10] Rosenwaike I, Shai D (1986) Trends in cancer mortality among Puerto Rican-born migrants to New York City. Int J Epidemiol 15: 30–35. 3957540 10.1093/ije/15.1.30

[11] Statistics Sweden (2006) Historic population register. 9–10.

[12] Socialstyrelsen Available:http://www.socialstyrelsen.se/register/dodsorsaksregistret. Accessed 4 July 2013.

[13] Socialstyrelsen Available: http://www.socialstyrelsen.se/Lists/Artikelkatalog/Attachments/18623/2012-3-6.pdf. Accessed 18 February 2013.

[14] Talback M, Stenbeck M, Rosen M, Barlow L, Glimelius B (2003) Cancer survival in Sweden 1960–1998–developments across four decades. Acta Oncologica 42: 637–659. 14690151 10.1080/02841860310013391

[15] Weires M, Bermejo JL, Sundquist K, Sundquist J, Hemminki K (2008) Socio-economic status and overall and cause-specific mortality in Sweden. BMC Public Health 8: 340 10.1186/1471-2458-8-340 18826562 PMC2564940

[16] The Swedish Register of Education Available: http://www.scb.se/statistik/UF/UF0506/Produktbeskrivning_short_English_UF0506_20040101r.doc. Accessed 8 November 2013.

[17] Socialstyrelsen Cancer Incidence in Sweden 2010. Available: http://www.socialstyrelsen.se/publikationer2011/2011-12-15. Accessed 14 October 2013.

[18] Johansson LA, Bjorkenstam C, Westerling R (2009) Unexplained differences between hospital and mortality data indicated mistakes in death certification: an investigation of 1,094 deaths in Sweden during 1995. J Clin Epidemiol 62: 1202–1209. 19364635 10.1016/j.jclinepi.2009.01.010

[19] Smith D, Taylor R, Coates M (1996) Socioeconomic differentials in cancer incidence and mortality in urban New South Wales, 1987–1991. Aust N Z J Public Health 20: 129–137. 8799086 10.1111/j.1753-6405.1996.tb01806.x

[20] Puigpinos R, Borrell C, Antunes JL, Azlor E, Pasarin MI, et al (2009) Trends in socioeconomic inequalities in cancer mortality in Barcelona: 1992–2003. BMC Public Health 9: 35 10.1186/1471-2458-9-35 19166582 PMC2640474

[21] Uthman OA, Jadidi E, Moradi T (2013) Socioeconomic position and incidence of gastric cancer: a systematic review and meta-analysis. J Epidemiol Community Health 67 In press. 10.1136/jech-2012-201108 23929615

[22] Ito S, Takachi R, Inoue M, Kurahashi N, Iwasaki M, et al (2008) Education in relation to incidence of and mortality from cancer and cardiovascular disease in Japan. Eur J Public Health 18: 466–472. 18628318 10.1093/eurpub/ckn052

[23] Fernandez E, Borrell C (1999) Cancer mortality by educational level in the city of Barcelona. Br J Cancer 79: 684–689. 10027350 10.1038/sj.bjc.6690108 PMC2362440

[24] Torres-Cintron M, Ortiz AP, Ortiz-Ortiz KJ, Figueroa-Valles NR, Perez-Irizarry J, et al (2012) Using a socioeconomic position index to assess disparities in cancer incidence and mortality, Puerto Rico, 1995–2004. Prev Chronic Dis 9: E15 10.5888/pcd9.100271 22172182 PMC3298767

[25] Wild SH, Fischbacher CM, Brock A, Griffiths C, Bhopal R (2006) Mortality from all cancers and lung, colorectal, breast and prostate cancer by country of birth in England and Wales, 2001–2003. Br J Cancer 94: 1079–1085. 16523198 10.1038/sj.bjc.6603031 PMC2361230

[26] Grulich AE, Swerdlow AJ, Head J, Marmot MG (1992) Cancer mortality in African and Caribbean migrants to England and Wales. Br J Cancer 66: 905–911. 1419634 10.1038/bjc.1992.383 PMC1977983

[27] Harding S, Rosato M, Teyhan A (2009) Trends in cancer mortality among migrants in England and Wales, 1979–2003. Eur J Cancer 45: 2168–2179. 19349162 10.1016/j.ejca.2009.02.029

[28] Regidor E, de La Fuente L, Martinez D, Calle ME, Dominguez V (2008) Heterogeneity in cause-specific mortality according to birthplace in immigrant men residing in Madrid, Spain. Ann Epidemiol 18: 605–613. 18652978 10.1016/j.annepidem.2008.04.007

[29] Bos V, Kunst AE, Keij-Deerenberg IM, Garssen J, Mackenbach JP (2004) Ethnic inequalities in age- and cause-specific mortality in The Netherlands. Int J Epidemiol 33: 1112–1119. 15166193 10.1093/ije/dyh189

[30] Mallin K, Anderson K (1988) Cancer mortality in Illinois Mexican and Puerto Rican immigrants, 1979–1984. Int J Cancer 41: 670–676. 3366488 10.1002/ijc.2910410506

[31] Spallek J, Arnold M, Razum O, Juel K, Rey G, et al (2012) Cancer mortality patterns among Turkish immigrants in four European countries and in Turkey. Eur J Epidemiol 27: 915–921. 23179631 10.1007/s10654-012-9746-y

[32] Razum O, Zeeb H, Akgun HS, Yilmaz S (1998) Low overall mortality of Turkish residents in Germany persists and extends into a second generation: merely a healthy migrant effect? Trop Med Int Health 3: 297–303. 9623931 10.1046/j.1365-3156.1998.00233.x

[33] Bouchardy C, Parkin DM, Khlat M (1994) Cancer mortality among Chinese and South-East Asian migrants in France. Int J Cancer 58: 638–643. 8077046 10.1002/ijc.2910580504

[34] Bouchardy C, Parkin DM, Wanner P, Khlat M (1996) Cancer mortality among north African migrants in France. Int J Epidemiol 25: 5–13. 8666504 10.1093/ije/25.1.5

[35] Rosenwaike I (1988) Cancer mortality among Mexican immigrants in the United States. Public Health Rep 103: 195–201. 3128838 PMC1477965

[36] Bouchardy C, Wanner P, Parkin DM (1995) Cancer mortality among sub-Saharan African migrants in France. Cancer Causes Control 6: 539–544. 8580303 10.1007/BF00054163

[37] Khlat M (1995) Cancer in Mediterranean migrants–based on studies in France and Australia. Cancer Causes Control 6: 525–531. 8580301 10.1007/BF00054161

[38] Nasseri K, Moulton LH (2011) Patterns of death in the first and second generation immigrants from selected Middle Eastern countries in California. J Immigr Minor Health 13: 361–370. 19621261 10.1007/s10903-009-9270-7

[39] Matos EL, Khlat M, Loria DI, Vilensky M, Parkin DM (1991) Cancer in migrants to Argentina. Int J Cancer 49: 805–811. 1959985 10.1002/ijc.2910490602

[40] McCredie M, Williams S, Coates M (1999) Cancer mortality in East and Southeast Asian migrants to New South Wales, Australia, 1975–1995. Br J Cancer 79: 1277–1282. 10098772 10.1038/sj.bjc.6690205 PMC2362226

[41] Balzi D, Geddes M, Brancker A, Parkin DM (1995) Cancer mortality in Italian migrants and their offspring in Canada. Cancer Causes Control 6: 68–74. 7718737 10.1007/BF00051682

[42] Public health in Stockholm County 2007. Report from Centre for Public Health. Stockholm; 2007. ISBN 978-91-975889-3-5.

[43] Ott JJ, Paltiel AM, Winkler V, Becher H (2008) Chronic disease mortality associated with infectious agents: a comparative cohort study of migrants from the Former Soviet Union in Israel and Germany. BMC Public Health 8: 110 10.1186/1471-2458-8-110 18400085 PMC2377256

[44] Ronellenfitsch U, Kyobutungi C, Ott JJ, Paltiel A, Razum O, et al (2009) Stomach cancer mortality in two large cohorts of migrants from the Former Soviet Union to Israel and Germany: are there implications for prevention? Eur J Gastroenterol Hepatol 21: 409–416. 19242359 10.1097/MEG.0b013e3283155220

[45] Swerdlow A (1991) Mortality and cancer incidence in Vietnamese refugees in England and Wales: a follow-up study. Int J Epidemiol 20: 13–19. 2066210 10.1093/ije/20.1.13

[46] Wang ZJ, Ramcharan S, Love EJ (1989) Cancer mortality of Chinese in Canada. Int J Epidemiol 18: 17–21. 2722361 10.1093/ije/18.1.17

[47] Kyobutungi C, Ronellenfitsch U, Razum O, Becher H (2006) Mortality from cancer among ethnic German immigrants from the Former Soviet Union, in Germany. Eur J Cancer 42: 2577–2584. 16914309 10.1016/j.ejca.2006.03.032

[48] Winkler V, Holleczek B, Stegmaier C, Becher H (2012) Prostate cancer in Germany among migrants from the Former Soviet Union. Glob Health Action 5: 9135 10.3402/gha.v5i0.9135 22229025 PMC3252511

[49] Beiki O, Ekbom A, Allebeck P, Moradi T (2009) Risk of prostate cancer among Swedish-born and foreign-born men in Sweden, 1961–2004. Int J Cancer 124: 1941–1953. 19107943 10.1002/ijc.24138

[50] Geddes M, Balzi D, Buiatti E, Khlat M, Parkin D (1991) Cancer in Italian migrants. Cancer Causes Control 2: 133–140. 1873437 10.1007/BF00053133

[51] Swerdlow AJ, Marmot MG, Grulich AE, Head J (1995) Cancer mortality in Indian and British ethnic immigrants from the Indian subcontinent to England and Wales. Br J Cancer 72: 1312–1319. 7577489 10.1038/bjc.1995.507 PMC2033952

[52] Beiki O, Hall P, Ekbom A, Moradi T (2012) Breast cancer incidence and case fatality among 4.7 million women in relation to social and ethnic background: a population-based cohort study. Breast Cancer Res 14: R5 10.1186/bcr3086 22225950 PMC3496120

[53] Yang D, Dzayee DA, Beiki O, de Faire U, Alfredsson L, et al (2012) Incidence and case fatality after day 28 of first time myocardial infarction in Sweden 1987–2008. Eur J Prev Cardiol 19: 1304–1315. 21965519 10.1177/1741826711425340

[54] Borne Y, Engstrom G, Essen B, Hedblad B (2012) Immigrant status and increased risk of heart failure: the role of hypertension and life-style risk factors. BMC Cardiovasc Disord 12: 20 10.1186/1471-2261-12-20 22443268 PMC3325899

[55] Seeff LC, McKenna MT (2003) Cervical cancer mortality among foreign-born women living in the United States, 1985 to 1996. Cancer Detect Prev 27: 203–208. 12787727 10.1016/s0361-090x(03)00062-x

[56] Azerkan F, Sparen P, Sandin S, Tillgren P, Faxelid E, et al (2012) Cervical screening participation and risk among Swedish-born and immigrant women in Sweden. Int J Cancer 130: 937–947. 21437898 10.1002/ijc.26084

