# Correction: Cancer Mortality by Country of Birth, Sex, and Socioeconomic Position in Sweden, 1961–2009

**DOI:** 10.1371/journal.pone.0106167

**Published:** 2014-08-13

**Authors:** The PLOS ONE Staff

There is an error in the legend for Figure 3. There are three question marks at the beginning of the legend which should instead be an asterisk. Please see the corrected Figure 3 here.

**Figure 3:**
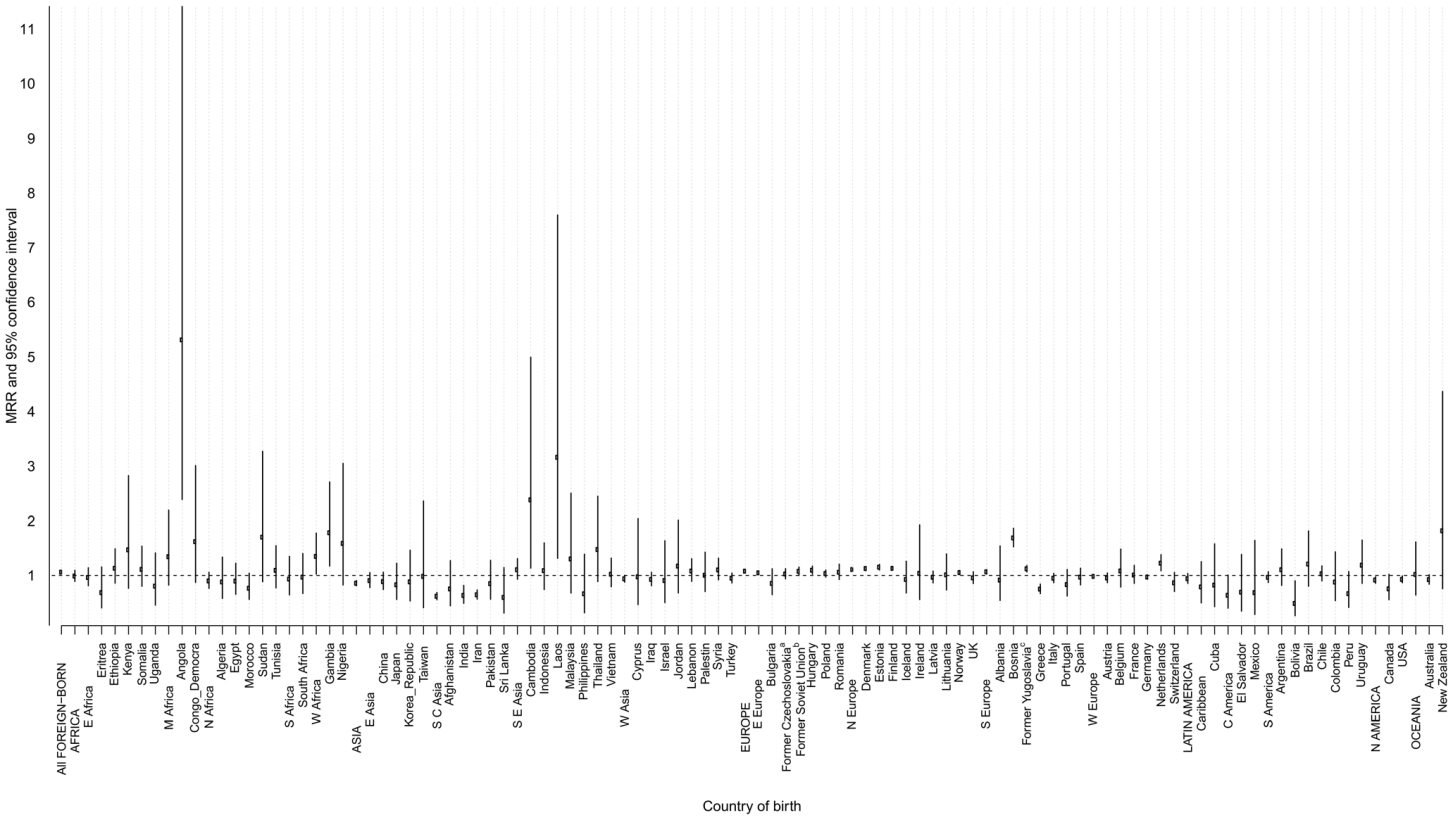
All-site cancer mortality rate ratio (MRR)* and 95% confidence interval (CI) among foreign-born men by continent, region, and country of birth compared with Sweden-born men, 1961–2009. *Adjusted for age at follow-up and calendar period at baseline. ^a^The former Czechoslovakia includes Czechoslovakia, Slovakia, and the Czech Republic. ^b^The former Soviet Union includes Belarus, Moldova, Russian Federation, Soviet Union, and Ukraine. ^c^The former Yugoslavia includes Yugoslavia, Croatia, Macedonia, Serbia, Slovenia, and Montenegro.
