# Supplementary material for: Cancer Mortality by Country of Birth, Sex, and Socioeconomic Position in Sweden, 1961–2009
Source: PLoS One. 2014 Mar 28;9(3):e93174. doi: 10.1371/journal.pone.0093174 (PMC3969357; doi:10.1371/journal.pone.0093174)
Supplement: Table S1 — All-site cancer mortality rate ratios (MRR) and 95% confidence interval (CI) in men by continent, region, and country of birth in Sweden, 1961–2009. *Mortality rate ratios (MRRs) are adjusted for age at follow-up and calendar period at baseline. The reference group is Sweden-born men. MRR values significantly different from 1.0 are highlighted in bold. **Continents, regions, and countries with at least five cases of cancer mortality. aThe former Czechoslovakia includes Czechoslovakia, Slovakia, and the Czech Republic. bThe former Soviet Union includes Belarus, Moldova, Russian Federation, Soviet Union, and Ukraine. cThe former Yugoslavia includes Yugoslavia, Croatia, Macedonia, Serbia, Slovenia, and Montenegro. (DOC) [file pone.0093174.s001.doc]

Table S1: All-site cancer mortality rate ratios (MRR) and 95% confidence interval (CI) in men by continent, region, and country of birth in Sweden, 1961–2009.

| **Country of birth**** | | | **Cases** | **Person-years** | **MRR* (95%CI)** | **Country of birth** | | | **Cases** | **Person-years** | **MRR* (95%CI)** |
| --- | --- | --- | --- | --- | --- | --- | --- | --- | --- | --- | --- |
|  | | |  |  |  |  | | |  |  |  |
| **Sweden** | | | 435548 | 187717196 | 1 |  | | **Northern Europe** | 16042 | 6789090 | **1.10 (1.08-1.12)** |
| **Outside Sweden** | | | 29142 | 16782285 | **1.05 (1.04-1.07)** |  | | Denmark | 3294 | 1041655 | **1.12 (1.08-1.16)** |
|  | **Africa** | | 373 | 738751 | 0.98 (0.89-1.09) |  | | Estonia | 1920 | 335477 | **1.14 (1.09-1.20)** |
|  | | **Eastern Africa** | 134 | 383898 | 0.96 (0.80-1.14) |  | | Finland | 7552 | 4141703 | **1.12 (1.10-1.15)** |
|  | | Eritrea | 14 | 52621 | 0.68 (0.40-1.15) |  | | Iceland | 40 | 56686 | 0.92 (0.67-1.26) |
|  | | Ethiopia | 51 | 129113 | 1.13 (0.85-1.48) |  | | Ireland | 10 | 16416 | 1.03 (0.55-1.92) |
|  | | Kenya | 9 | 13601 | 1.46 (0.76-2.82) |  | | Latvia | 307 | 62455 | 0.96 (0.86-1.07) |
|  | | Somalia | 37 | 130794 | 1.10 (0.80-1.53) |  | | Lithuania | 37 | 15542 | 1.01 (0.73-1.39) |
|  | | Uganda | 12 | 29255 | 0.80 (0.45-1.41) |  | | Norway | 2581 | 844014 | **1.05 (1.01-1.09)** |
|  | | **Middle Africa** | 16 | 25122 | 1.34 (0.82-2.19) |  | | UK | 301 | 275137 | 0.95 (0.85-1.06) |
|  | | Angola | 6 | 4202 | **5.31 (2.38-11.8)** |  | | **Southern Europe** | 3111 | 2351490 | **1.06 (1.02-1.10)** |
|  | | Congo_Democratic | 10 | 12838 | 1.61 (0.87-3.00) |  | | Albania | 14 | 8552 | 0.91 (0.54-1.54) |
|  | | **North Africa** | 144 | 214277 | 0.89 (0.76-1.05) |  | | Bosnia | 637 | 412281 | **1.68 (1.52-1.86)** |
|  | | Algeria | 22 | 30941 | 0.87 (0.57-1.33) |  | | Former Yugoslavia^c^ | 1508 | 1205919 | **1.12 (1.06-1.18)** |
|  | | Egypt | 39 | 38891 | 0.89 (0.65-1.22) |  | | Greece | 283 | 323539 | **0.74 (0.66-0.84)** |
|  | | Morocco | 40 | 75960 | 0.76 (0.55-1.04) |  | | Italy | 468 | 218452 | 0.94 (0.86-1.03) |
|  | | Sudan | 9 | 9686 | 1.70 (0.88-3.26) |  | | Portugal | 46 | 53915 | 0.83 (0.62-1.11) |
|  | | Tunisia | 32 | 51766 | 1.09 (0.77-1.54) |  | | Spain | 154 | 127257 | 0.96 (0.82-1.13) |
|  | | **Southern Africa** | 28 | 22742 | 0.93 (0.64-1.35) |  | | **Western Europe** | 3246 | 1316145 | 0.98 (0.94-1.01) |
|  | | South Africa | 28 | 21060 | 0.96 (0.66-1.40) |  | | Austria | 433 | 184573 | 0.95 (0.86-1.04) |
|  | | **Western Africa** | 51 | 92710 | **1.34 (1.02-1.77)** |  | | Belgium | 38 | 20217 | 1.08 (0.78-1.48) |
|  | | Gambia | 22 | 32784 | 1.78 (1.17-2.70) |  | | France | 143 | 99194 | 1.00 (0.85-1.18) |
|  | | Nigeria | 9 | 15218 | 1.58 (0.82-3.04) |  | | Germany | 2274 | 853316 | 0.96 (0.93-1.01) |
|  | **Asia** | | 1661 | 3021449 | **0.85 (0.80-0.90)** |  | | Netherlands | 263 | 103474 | **1.22 (1.08-1.38)** |
|  | | **Eastern Asia** | 170 | 220317 | 0.90 (0.77-1.05) |  | | Switzerland | 94 | 53629 | 0.86 (0.70-1.05) |
|  | | China | 122 | 88153 | 0.88 (0.74-1.05) |  | **Latin America** | | 426 | 734473 | 0.94 (0.85-1.03) |
|  | | Japan | 25 | 27799 | 1.11 (0.75-1.65) |  | | **Caribbean** | 18 | 29622 | 0.78 (0.49-1.25) |
|  | | Korea_Republic | 15 | 92404 | 0.88 (0.53-1.46) |  | | Cuba | 9 | 14898 | 0.82 (0.42-1.57) |
|  | | Taiwan | 5 | 6338 | 0.98 (0.40-2.35) |  | | **Central America** | 18 | 59094 | 0.63 (0.39-1.00) |
|  | | **South_Central Asia** | 362 | 976053 | **0.66 (0.59-0.74)** |  | | El Salvador | 8 | 25655 | 0.69 (0.34-1.38) |
|  | | Afghanistan | 14 | 53182 | 0.75 (0.44-1.27) |  | | Mexico | 5 | 12941 | 0.68 (0.28-1.64) |
|  | | India | 58 | 136401 | **0.63 (0.48-0.81)** |  | | **South America** | 390 | 645756 | 0.96 (0.87-1.06) |
|  | | Iran | 253 | 621719 | **0.64 (0.56-0.73)** |  | | Argentina | 43 | 40749 | 1.10 (0.81-1.48) |
|  | | Pakistan | 23 | 54098 | 0.84 (0.56-1.27) |  | | Bolivia | 10 | 33360 | **0.48 (0.26-0.90)** |
|  | | Sri Lanka | 9 | 63052 | 0.59 (0.30-1.14) |  | | Brazil | 23 | 35455 | 1.20 (0.80-1.81) |
|  | | **South_Eastern Asia** | 135 | 269808 | **0.61 (0.55-0.68)** |  | | Chile | 234 | 339096 | 1.02 (0.90-1.17) |
|  | | Cambodia | 7 | 3967 | **2.37 (1.13-4.99)** |  | | Colombia | 16 | 91958 | 0.87 (0.53-1.43) |
|  | | Indonesia | 26 | 26624 | 1.08 (0.74-1.59) |  | | Peru | 17 | 44619 | 0.66 (0.41-1.07) |
|  | | Laos | 5 | 3488 | **3.15 (1.31-7.59)** |  | | Uruguay | 36 | 35601 | 1.18 (0.85-1.64) |
|  | | Malaysia | 9 | 13687 | 1.30 (0.67-2.50) |  | **North America** | | 885 | 340585 | **0.91 (0.85-0.97)** |
|  | | Philippines | 7 | 28665 | 0.66 (0.31-1.38) |  | | Canada | 41 | 37624 | 0.75 (0.55-1.02) |
|  | | Thailand | 15 | 69397 | 1.47 (0.88-2.44) |  | | USA | 844 | 302260 | **0.92 (0.86-0.99)** |
|  | | Vietnam | 61 | 116740 | 1.02 (0.79-1.31) |  | **Oceania** | | 18 | 41072 | 1.01 (0.64-1.61) |
|  | | **Western Asia** | 994 | 1555270 | 0.93 (0.87-1.00) |  | | **Australia_New** | 18 | 40176 | 1.04 (0.66-1.66) |
|  | |  |  |  |  |  | | **Zealand** |  |  |  |
|  | | Cyprus | 7 | 9851 | 0.97 (0.46-2.04) |  | | Australia | 13 | 31297 | 0.92 (0.53-1.58) |
|  | | Iraq | 245 | 568402 | 0.92 (0.80-1.05) |  | | New Zealand | 5 | 8878 | 1.81 (0.75-4.36) |
|  | | Israel | 11 | 26344 | 0.90 (0.50-1.63) |  | |  |  |  |  |
|  | | Jordan | 13 | 18731 | 1.16 (0.67-2.01) |  | |  |  |  |  |
|  | | Lebanon | 109 | 242658 | 1.07 (0.89-1.30) |  | |  |  |  |  |
|  | | Palestinian territories | 31 | 23397 | 1.00 (0.70-1.42) |  | |  |  |  |  |
|  | | Syrian Arab Republic | 124 | 158199 | 1.10 (0.91-1.31) |  | |  |  |  |  |
|  | | Turkey | 446 | 473671 | 0.94 (0.86-1.04) |  | |  |  |  |  |
|  | **Europe** | | 25697 | 11854841 | **1.07 (1.06-1.09)** |  | |  |  |  |  |
|  | | **Eastern Europe** | 3298 | 1398115 | **1.04 (1.01-1.08)** |  | |  |  |  |  |
|  | | Bulgaria | 51 | 46396 | 0.85 (0.64-1.12) |  | |  |  |  |  |
|  | | Former Czechoslovakia^a^ | 466 | 169341 | 1.02 (0.93-1.12) |  | |  |  |  |  |
|  | | Former Soviet Union^b^ | 736 | 202551 | 1.07 (0.99-1.15) |  | |  |  |  |  |
|  | | Hungary | 842 | 341727 | **1.09 (1.02-1.17)** |  | |  |  |  |  |
|  | | Poland | 979 | 499680 | 1.03 (0.96-1.10) |  | |  |  |  |  |
|  | | Romania | 224 | 138418 | 1.05 (0.92-1.20) |  | |  |  |  |  |

*Mortality rate ratios (MRRs) are adjusted for age at follow-up and calendar period at baseline. The reference group is Sweden-born men. MRR values significantly different from 1.0 are highlighted in bold.

**Continents, regions, and countries with at least five cases of cancer mortality.

^a^The former Czechoslovakia includes Czechoslovakia, Slovakia, and the Czech Republic.

^b^The former Soviet Union includes Belarus, Moldova, Russian Federation, Soviet Union, and Ukraine.

^c^The former Yugoslavia includes Yugoslavia, Croatia, Macedonia, Serbia, Slovenia, and Montenegro.
